# Intersectoral Collaboration Between Educational and Mental Health Services for Autistic Children and Adolescents in Brazil

**DOI:** 10.3390/healthcare14091170

**Published:** 2026-04-27

**Authors:** Leni Porto Costa Siqueira, Valentina Acosta Bermúdez, Valentina Franco Gomes, Guilherme Carvalho de Paula Francisco, Felipe Alckmin-Carvalho, Piyali Bhattacharya, Andrew D. R. Surtees, Maria Cristina Triguero Veloz Teixeira

**Affiliations:** 1 Postgraduate Program on Human Developmental Sciences, Mackenzie Center for Research in Childhood and Adolescence, Mackenzie Presbyterian University, São Paulo 01302-907, SP, Brazil; lenitutora@gmail.com (L.P.C.S.); valentina.bermudez@mackenzista.com.br (V.A.B.); guilherme_atas@hotmail.com (G.C.d.P.F.); 2 Postgraduate Program in Clinical and Health Psychology, Department of Psychology and Education, Faculty of Social and Human Sciences, University of Beira Interior, 6200-209 Covilhã, Portugal; felipe.carvalho@ubi.pt; 3 Research Center in Sports Sciences, Health Sciences and Human Development (CIDESD-UBI), University of Beira Interior, 6201-001 Covilhã, Portugal; 4 School of Psychology, University of Birmingham, Edgbaston, Birmingham B15 2TT, UK; piya.bhattacharya4@gmail.com (P.B.); a.surtees@bham.ac.uk (A.D.R.S.)

**Keywords:** autism spectrum disorder, intersectoral collaboration, education, mental health

## Abstract

Introduction/Objectives: Intersectoral collaboration between education and mental health services is central to the care of autistic children and adolescents. However, recent literature indicates that evidence remains limited regarding how these collaborative arrangements are implemented in routine public services, particularly in low- and middle-income countries (LMICs). This study aimed to assess the intersectoral collaboration between Brazilian educational and mental health services for autistic children and adolescents and to examine the frequency and type of intersectoral contact. Methods: An exploratory cross-sectional study was conducted in the municipal public education system of Niterói, a city in the Southeast region of Brazil. Participants included parents of 123 autistic children and adolescents, 49 teachers from mainstream education and specialized educational services (SES), and 24 health professionals. Data were collected using structured questionnaires with multi-informant reports. The instruments were specifically developed for the study and submitted to expert content-validation procedures. Analyses included descriptive statistics and, in a subsample of 51 matched cases with paired responses from teachers and health professionals, Cohen’s kappa to assess agreement between reports. Results: Low levels of intersectoral collaboration were observed, characterized by infrequent contact, limited information exchange, and slight agreement between reports from teachers and health professionals (κ = 0.25; *p* = 0.01). Teachers were more likely to know where care was provided than to know which specialists were involved, while more than half of health professionals did not know which school the child attended. Conclusions: In the investigated municipal network, care appeared fragmented, highlighting difficulties in translating intersectoral recommendations from public policies into routine collaborative practices.

## 1. Introduction

Autism spectrum disorder (ASD) is a neurodevelopmental disorder [1] that often requires long-term follow-up after early diagnosis [2] and support across health, education, and family contexts [3,4]. Recent high-impact reviews emphasize that the quality of autism care depends not only on the availability of evidence-based interventions, but also on the capacity of services to coordinate goals, information, and responsibilities across settings [3,5]. When mental health and educational professionals act independently, care can become fragmented, leading to duplicated efforts, inconsistent strategies, and gaps in follow-up [5,6,7]. These challenges tend to be amplified in low- and middle-income countries (LMICs), where service availability and continuity are already uneven [8,9].

Recognized terms for these collaborative actions are intersectoral collaboration [10] and multi-agency working [11]. In this study, these terms refer to coordinated practices between sectors that include reciprocal knowledge of the services involved in the same case, communication between professionals, exchange of reports, and shared responsibility for care planning [12,13]. Brazil is an LMIC marked by territorial inequalities in the availability of services for autistic children and adolescents [14]. Evidence shows a concentration of specialized services in the Southeast and South regions, with substantially lower coverage in other parts of the country [15,16]. This distribution means that the formal existence of policies does not automatically translate into coordinated access or continuity of care in routine practice [16,17].

Brazilian legislation adopts intersectorality as a principle for reducing fragmentation between health and education and for organizing care through service networks [18]. National guidelines for autistic children explicitly recommend coordination between the education and health sectors, particularly for early identification, intervention planning, and long-term follow-up [19,20]. The Guidelines for Rehabilitation Care for Autistic Individuals recommend the development of an Individualized Therapeutic Project (Projeto Terapêutico Singular—PTS) based on coordinated actions among health professionals, educators, and families [19]. However, despite this normative framework, evidence on how these recommendations are translated into school practice remains limited [21].

Recent reviews have reinforced the relevance of coordinated care, while also showing that important knowledge gaps remain. An umbrella review focused on autism care in low- and middle-income countries identified fragmented or uncoordinated services as a recurrent barrier to universal health coverage [9]. Likewise, recent work has argued that integrated care pathways are necessary precisely because autism support typically spans multiple services and developmental contexts [3,5,12]. Nevertheless, much of the available literature focuses on access to diagnosis or intervention, rather than on whether education and mental health professionals involved with the same child actually communicate and share information in everyday public-service settings. Thus, the gap addressed by the present study is not whether intersectoral collaboration is recommended, but whether it occurs in practice, how frequently it occurs, and whether professionals from different sectors provide convergent accounts of the same collaborative actions. Accordingly, this study aimed to assess the intersectoral collaboration between Brazilian educational and mental health services for autistic children and adolescents. Additionally, the frequency and type of intersectoral collaboration were examined.

## 2. Materials and Methods

### 2.1. Ethical Considerations

The study was approved by the Research Ethics Committee for Human Subjects of Mackenzie Presbyterian University under protocol number 12831519.9.0000.0084. All ethical principles for research involving human beings were observed, in accordance with the Declaration of Helsinki.

### 2.2. Study Design and Setting

This exploratory cross-sectional study was conducted between August 2020 and January 2021 in the municipal public basic education system of Niterói, Rio de Janeiro, Brazil. A non-probabilistic sampling strategy was used because the study aimed to map how intersectoral collaboration was operating in routine public services at a defined period and to compare reports from different informants involved in the same cases. This design was appropriate for describing current patterns of communication and information exchange; however, it does not allow causal inferences or assessment of change over time. The municipality has 49 public schools serving students from the 1st to the 9th grade of elementary education. In Brazil, elementary education is divided into two stages: grades 1–5 (ages 6–10 years) and grades 6–9 (ages 11–14 years). All school administrative teams were contacted. Of the 49 schools, 45 reported a total of 217 autistic students. These students constituted the eligible study population. All identified students received support through Specialized Educational Services (SES) and mental health care provided by public services integrated into the Psychosocial Care Network (PCN), including a Child and Adolescent Psychosocial Care Center and specialized multiprofessional rehabilitation centers.

### 2.3. Participants

The study included parents/caregivers, teachers from municipal public educational services, and health professionals who provided care to autistic children and adolescents in southeastern Brazil. Of the 45 schools enrolling autistic children and adolescents, two did not authorize participation, and four were unable to participate because data collection, scheduled to begin in August 2020, was interrupted by school closures during the COVID-19 pandemic. The study included 39 schools (86.6%) from the municipality, with 217 eligible autistic students, and, of the 217 eligible students, 123 parent questionnaires were obtained, corresponding to 56.6% of the eligible children/adolescents; 43.3% did not participate in the study. Of the total of 123 students, one parent reported on three autistic children, and another reported on two. Therefore, 123 child/adolescent cases corresponded to 118 adult parent/caregiver informants. This low participation was primarily attributable to the context of the COVID-19 pandemic. Four schools did not grant authorization for data collection involving parents, while in the remaining schools, significant challenges in reaching parents by telephone limited participation, ultimately precluding data collection during this period. The mean age of parents was 40.3 years (SD = 9.0), and 88.6% were biological parents. More than half had completed high school (53.3%), and most were classified as middle-low income (46.6%), with 69.1% reporting not receiving government assistance benefits (Table 1 and Table 2). Teacher questionnaires were obtained from 49 female teachers (mean age = 50.6 years; SD = 7.8), many of whom worked in mainstream education and/or SES and were directly involved in inclusion processes. Health professional data were obtained from 24 professionals (mean age = 42.2 years; SD = 9.6) from psychology, speech therapy, occupational therapy, social work, music therapy, and pedagogy-linked home care, reflecting the multiprofessional composition of the municipal network (Table 1). The unit of analysis for family and child contextual data was the child/adolescent case (*n* = 123). For intersectoral agreement analysis, the relevant unit was the matched case (*n* = 51), defined as a child/adolescent whose parents consented, and the teacher and the healthcare professional provided information. Of the 123 children and adolescents, we obtained responses from professionals for only 41.4% of cases, corresponding to a sample loss of 58.5%. The average age of the children/adolescents was 10 years (SD = 3.1), with a predominance of males (86.2%). Most were enrolled in Elementary Education, level I (81.3%). Regarding age at diagnosis, 74.7% were diagnosed between 12 and 48 months of age, whereas 21.9% received the diagnosis between 5 and 8 years of age. Parent data were used in specific analyses related to family characteristics, caregiving conditions, and contextual aspects (Table 2).

### 2.4. Instruments

The three questionnaires were specifically developed for this study to capture complementary and comparable indicators of intersectoral collaboration across informants. Item development was guided by the study objectives, Brazilian policy documents on autism care and intersectorality [18,19], and prior literature on service access and coordination in autism [9,16]. Because the instruments were designed to describe heterogeneous service-process indicators rather than a single latent construct, the psychometric strategy prioritized evidence of content validity rather than internal consistency. Development involved drafting items, expert review by three professionals with expertise in neurodevelopmental disorders, and wording refinement before field application [22].

Sociodemographic and clinical questionnaire: This form assessed the sociodemographic characteristics of the families of children and adolescents, such as sex, relationship with the child, educational level, family composition, number of siblings of the child, and socioeconomic level. The clinical data collected about the child included the diagnosis of ASD, age, sex, age at diagnosis, educational level, type of healthcare professionals attending the child and monthly frequency of appointments, type of institution where the child receives healthcare, and types of interventions received from healthcare professionals. The form included items assessing parent-reported educational information, including curricular adaptations and/or accommodations; participation in special education services during regular school hours or after-school periods; types of stimulation received; changes in academic skills over the past 12 months; the presence of educational support or a therapeutic aide at school; and the type, frequency, and content of contacts between the school and the municipal mental health services responsible for the child’s care. This form also included items assessing parent-reported mental health information, including the frequency and type of mental health services; procedures and interventions used; medication use; the type, frequency, and content of contacts between mental health services and schools; and changes in the child’s mental health and adaptive functioning over the past 12 months. The questionnaire items were scored using either frequency-based scales or dichotomous (yes/no) response options (Supplementary Material File S1). The form was reviewed by three professionals with expertise in neurodevelopmental disorders, who evaluated all items according to objectivity, clarity, and precision criteria [22]. Content validity coefficients were 0.91 for objectivity, 0.93 for clarity, and 0.90 for precision, indicating excellent content validity.

Educational services form: This instrument was developed to evaluate the special education services delivered to autistic children in school settings and was completed by teachers. It included items on academic domains such as curricular adaptations and/or accommodations; participation in special education services during regular school hours or after-school periods; types of stimulation received; changes in academic skills over the past 12 months; educational support or a therapeutic aide at school; and the type, frequency, and content of contacts between the school and the municipal mental health services responsible for the child’s care. The questionnaire items were scored using either frequency-based scales or dichotomous (yes/no) response options (Supplementary Material File S2). The form was reviewed by three professionals with expertise in neurodevelopmental disorders, who evaluated all items according to objectivity, clarity, and precision criteria [22]. Content validity coefficients were 0.78 for objectivity, 0.83 for clarity, and 0.80 for precision, indicating acceptable-to-good content validity.

Mental health services form: This instrument was developed to evaluate the mental health services delivered to autistic children and was completed by health professionals. It included items on mental health domains such as the composition of the multidisciplinary healthcare team; the frequency and modality of services; procedures and interventions used; medication use; the type, frequency, and content of contacts between mental health services and schools; and changes in the child’s mental health and adaptive functioning over the past 12 months. The questionnaire items were scored using either frequency-based scales or dichotomous (yes/no) response options (Supplementary Material File S3). The form was reviewed by three professionals with expertise in neurodevelopmental disorders, who evaluated all items according to objectivity, clarity, and precision criteria [22]. Content validity coefficients were 0.78 for objectivity, 0.83 for clarity, and 0.83 for precision, indicating acceptable-to-good content validity.

### 2.5. Data Collection Procedures

The data were collected via video calls during the COVID-19 pandemic. The participants were instructed to report their responses with reference to the pre-pandemic period in order to minimize potential biases related to school closures and disruptions in mental health services. Teachers completed the educational services form at school, and health professionals completed the mental health services form at their respective public services (the Monteiro Lobato Child and Adolescent Psychosocial Care Center and the Specialized Rehabilitation Centers: the Fluminense Association of Rehabilitation, the Association for the Support of the Blind, and the Pestalozzi Association of Niterói). The study sought paired teacher and health professional data for the same child/adolescent whenever both sectors could be reached within the public network. Health professionals completed forms for 51 children/adolescents, generating the matched subsample used for agreement analyses. Teachers and health professionals responded independently and were blinded to each other’s answers. Losses in the matched subsample were related to discharge from services, absence of records documenting the care provided, and treatment dropout.

### 2.6. Data Analysis

Analyses were conducted using IBM SPSS Statistics for Windows, Version 20.0 (IBM Corp., Armonk, NY, USA). First, descriptive statistics (absolute frequencies, percentages, means, and standard deviations) were used to summarize participant characteristics and indicators of intersectoral collaboration. The broader parental sample (*n* = 123) informed the family and contextual characterization, whereas the matched subsample (*n* = 51) was used for cross-informant analyses involving teachers and health professionals. For the interpretation of the Content Validity Index of the forms developed for parents, teachers, and health professionals, the following criteria were adopted: acceptable (0.78–0.79), good (0.80–0.89), and excellent (≥0.90) [22]. Agreement between teacher and health professional reports was examined using Cohen’s kappa (κ) for the corresponding categorical item on guidance about treatment progress/interventions exchanged between services. Only paired responses referring to the same child/adolescent were included in this calculation, and missing responses were retained as missing (i.e., not imputed). Kappa values were interpreted as follows: <0.20 (poor agreement), 0.21–0.40 (slight), 0.41–0.60 (moderate), 0.61–0.80 (substantial), and 0.81–1.00 (almost perfect agreement) [23]. The significance level adopted was 5% (*p* < 0.05). Because the study was exploratory and descriptive, no multivariable modeling was planned.

## 3. Results

### Intersectoral Collaboration and Information Exchange

As shown in Table 3, intersectoral collaboration between educational and mental health services revealed limited and asymmetrical cross-sectoral awareness. Teachers were more likely to know where mental health care was provided (88.2%) and whether the child used medication (72.5%) than to know which specialists were responsible for care (11.8%) or which procedures/interventions were being conducted (41.2%). Health professionals, in turn, frequently lacked information about the educational context: 56.8% did not know which school the child attended, and 45.0% were unaware of the support implemented by the pedagogical and educational support teacher.

Table 4 shows that reported contacts between sectors were infrequent and irregular. Teachers most commonly reported contacting health professionals every six months (50.9%), whereas health professionals most frequently reported annual contact (47.0%). For contacts initiated by health professionals, 47.0% of health professionals and 64.7% of teachers reported no interservice contact.

Table 4 also indicates an asymmetry in information exchange. Reports on students’ academic development were sent by teachers to health professionals in 54.9% of cases, whereas reports on intervention progress were sent by health professionals to teachers in 29.4% of cases. Agreement between teacher and health professional reports regarding guidance on treatment progress and interventions was slight, although statistically significant (κ = 0.25; *p* = 0.01). Taken together, these findings suggest that some communication occurred, but it was neither systematic nor sufficiently consistent to produce convergent reports across sectors.

## 4. Discussion

Our findings suggest that intersectoral collaboration in this municipal public network was not entirely absent, but it was sparse, uneven, and weakly structured. This pattern contrasts with Brazilian Ministry of Health guidelines, such as the “Guidelines for Rehabilitation Care for Individuals with Autism Spectrum Disorders”, which recommend collaborative actions, case discussion, and coordinated planning through the Individualized Therapeutic Project (PTS) and network-based care [19]. Recent literature has similarly emphasized that high-quality autism care depends on integrated pathways capable of connecting services across developmental contexts [3,5,12]. Limited to this sample, we found that professionals from education and mental health frequently lacked basic information about the counterpart service involved in the same case, suggesting that the gap between normative recommendations and routine practice may emerge at a very operational level.

A key finding was the asymmetry of what professionals knew about one another’s work. Teachers more often knew where care was provided and whether the child used medication than they knew which specialists were involved or what procedures/interventions were underway. Health professionals, in turn, often did not know the school in which the child was enrolled or the supports implemented in the educational setting. This pattern is relevant because coordinated care depends not only on service availability, but also on reciprocal knowledge that allows professionals to align goals, adapt strategies across contexts, and avoid contradictory guidance [3,5,9]. When symptoms or learning-related difficulties observed in one setting are not systematically communicated to the other, opportunities for shared problem-solving are reduced, and care tends to remain compartmentalized [24]. The low and inconsistent frequency of contact between the professionals in our sample may be one of the factors that reinforces this interpretation. Discrepancies in contact information emerged as one of the findings, indicating that inconsistencies in the frequency reported by participating professionals may negatively impact intersectoral actions.

Some teachers reported semiannual contact, and some health professionals reported annual contact, while a substantial proportion of respondents from both sectors described no contact at all. Moreover, the direction of information exchange was unequal: academic reports were more often sent from school to health services than treatment-progress reports were sent from health services to school. The statistically significant, yet slight, agreement (κ = 0.25) should therefore be interpreted as evidence of low convergence between professional reports, rather than as proof that collaboration never occurred. In practical terms, contact may have been occasional and case-specific but not sufficiently formalized to generate a shared understanding of what collaborative actions were actually taking place.

This interpretation is consistent with previous Brazilian evidence. Lima and colleagues [25], when studying the Psychosocial Care Network in the metropolitan region of Rio de Janeiro, found that coordination with education was frequently mentioned as an intersectoral objective, yet contacts with schools tended to occur on a case-by-case basis rather than through institutionalized partnerships. Our findings add to this literature by examining matched reports from teachers and health professionals involved with the same children/adolescents, thereby showing that fragmentation can be observed not only in policy implementation narratives, but also in the limited mutual awareness and low report convergence reported by frontline professionals.

The findings also help clarify an important theoretical point. The presence of multiple services in a child’s care pathway does not by itself indicate integrated care. Service availability and service integration are related but distinct dimensions [5,9,26]. In our study, children and adolescents were connected to both educational and mental health services, yet communication patterns remained weak.

This study did not investigate factors associated with the low levels of intersectoral collaboration reported by participating professionals. Nevertheless, it is plausible that some of these factors mirror those described in a recent study from Chile [12], which identified a lack of nationally recognized technical standards for intersectoral action in Chile, alongside persistent challenges in coordination—especially between the education and health sectors—resulting in fragmented support pathways and compromised continuity of care.

This distinction is particularly relevant in LMIC contexts, where expanding access is necessary but may still fall short of ensuring continuity, coordination, and comprehensiveness of care [9,26].

### 4.1. Practical and Theoretical Implications

From a practical perspective, despite the limitation that the data were derived from a single municipality in southeastern Brazil, the findings suggest that relatively low-cost organizational strategies may enhance intersectoral collaboration within municipal service networks. Potential strategies include the designation of a reference professional in each sector, the systematic use of child development indicators, the implementation of periodic case review meetings, and the routine sharing of core information on academic and mental health domains, among others. From a theoretical standpoint, the study contributes by operationalizing intersectoral collaboration through observable process indicators, mutual knowledge, contact frequency, information exchange, and cross-informant agreement rather than treating collaboration as a purely normative principle. Highlighting the importance of intersectoral actions, a recent study on the socioeconomic burden of autism caregiving in Africa drew on multiple databases and grey literature sources, including policy documents [10]. This study emphasized the need for coordinated intersectoral collaboration among health, education, social protection, and community sectors to effectively address the complexities of autism care. This helps bridge the gap between policy discourse and measurable features of everyday service delivery.

### 4.2. Limitations and Future Directions

This study has limitations that should be considered when interpreting the findings. First, the cross-sectional design offers only a snapshot of collaboration and does not allow causal inference or assessment of temporal changes. Second, data collection occurred during the COVID-19 pandemic; although participants were instructed to report on the pre-pandemic period, this retrospective reporting may have introduced recall bias. Third, the study was conducted in a single municipality and used a non-probabilistic sample, which limits generalizability to other Brazilian contexts. Fourth, although parent data were obtained for 123 cases, agreement analyses depended on a smaller matched subsample of 51 cases with paired professional responses, reducing the breadth of the cross-informant analysis. Fifth, non-participation and incomplete recruitment are common in naturalistic studies relying on voluntary consent, especially in contexts involving children and requiring the consent of legal parents/caregivers.

Furthermore, the final number of children/adolescents about whom health professionals and parents responded was small, as was the low participation of parents in the study (of the 217 eligible autistic students, information was only obtained for 123 children). Finally, although the instruments were designed to assess service process indicators and underwent content validation, they did not include direct observation of intersectoral practices, nor did they contain items aimed at investigating the reasons underlying the absence of such actions.

Future research would benefit from longitudinal and mixed-methods designs conducted in multiple municipalities, with direct examination of communication flows, organizational barriers, and the mechanisms that facilitate or hinder collaboration between sectors.

## 5. Conclusions

This study provides empirical evidence from a municipal public network of a single municipality in southeastern Brazil indicating that intersectoral collaboration between education and mental health services for autistic children and adolescents was infrequent, asymmetrical, and only weakly convergent across professional reports. Teachers and health professionals did not consistently know what support the other sector was providing, contact between sectors was irregular, and information exchange was more often initiated by schools than by mental health services.

These findings support the interpretation that care was fragmented in the investigated municipality, but they should not be read as a definitive characterization of all Brazilian contexts; rather, they highlight the need for future multi-site studies to expand on these findings. The study also shows that the existence of policy recommendations for intersectoral care does not guarantee their routine operationalization. Strengthening communication mechanisms, clarifying responsibilities across sectors, and formalizing case-based coordination may be important steps toward more integrated care for autistic children and adolescents.

## Figures and Tables

**Table 1:** Sociodemographic characterization of the adult participants (parents, teachers and health professionals).

Profile of the Participants			
Variables	Parents or Caregiver	Teachers	Health Professionals ^1^
Number of informants	118	49	24
Sex			
Male	7	0	3 (12.5)
Female	111	49 (100.0)	21 (87.5)
Mean age (Standard Deviation)	40.3 (9.01)	50.6 (7.8)	42.2 (9.6)
Educational level			
Elementary Education	44 (35.2)	0	0
High School	63 (53.3)	0	0
Undergraduate Degree	11 (9.3)	3 (6.1)	7 (29.1)
Postgraduate Certificate/Specialization	0	44 (89.7)	15 (62.5)
Postgraduate degree (Master’s or PhD)	0	2 (4.0)	2 (8.3)

Note: ^1^ Health professionals’ specialties (10 psychologists, 7 speech-language pathologists, 2 occupational therapists, 1 music therapist, 2 social workers, and 1 home caregiver with a degree in Pedagogy).

**Table 2:** Sociodemographic characterization of children and adolescents (*n* = 123).

Variables	Mean (Standard Deviation)/Frequency (%)
Age	10 (3.1)
Sex	
Male	106 (86.2)
Female	17 (13.8)
Educational level	
Elementary Education, level I (for children between 6 and 10 years)	100 (81.3)
Elementary Education, level II (for children between 11 and 14 years)	23 (18.7)
Age at diagnosis	
Between 12 and 24 months	43 (35.7)
Between 25 and 48 months	48 (39.0)
Between 5 and 8 years	27 (21.9)
Over 8 years	5 (4.0)
Household composition	
Biological mother/father and siblings	109 (88.6)
Adoptive mother/father and siblings	8 (6.5)
Grandmother/Grandfather	5 (4.1)
Other (Aunts, uncles, or other caregivers)	1 (0.8)
Socioeconomic level	
Higher income	3 (2.5)
Middle-High income	38 (32.2)
Middle-Low income	54 (46.6)
Low income	22 (18.6)

**Table 3:** Cross-sector knowledge of educational and mental health services reported by teachers and health professionals in the matched subsample (*n* = 51).

Information Assessed	Aware *n* (%)	Not Aware *n* (%)
Knowledge of teachers about mental health services received by the child (*n* = 51)		
Mental health service where the child receives care	45 (88.2)	6 (11.8)
Specialists who provide care for the child in the mental health service	6 (11.8)	45 (88.2)
Types of procedures/interventions received in the mental health service	21 (41.2)	30 (58.8)
Child’s use of medication	37 (72.5)	14 (27.5)
Knowledge of health professionals about the educational services received by the child (*n* = 51)		
School in which the child/adolescent is enrolled	22 (43.1)	29 (56.8)
Supports implemented by the pedagogical and educational support teacher	28 (54.9)	23 (45.0)

**Table 4:** Frequency of interservice contact and information exchange reported by teachers and health professionals in the matched subsample (*n* = 51).

Type of Contact/Information	Never *n* (%)	Every 6 Months *n* (%)	Every 12 Months *n* (%)
Contacts made by teachers with health professionals			
SES teachers’ report	18 (35.2)	26 (50.9)	7 (13.7)
Health professionals’ report	21 (41.1)	6 (11.7)	24 (47.0)
Contacts made by health professionals with teachers			
SES teachers’ report	33 (64.7)	2 (3.9)	16 (31.3)
Health professionals’ report	24 (47.0)	8 (15.6)	19 (37.2)
Interprofessional information exchange between services	Yes *n* (%)	No *n* (%)	
Reports sent by teachers (academic development) to health professionals	28 (54.9)	23 (45.0)	
Reports sent by health professionals (intervention progress) to teachers	15 (29.4)	14 (27.4)	

Note. SES = specialized educational service. Percentages are based on the 51 matched cases. Totals lower than 51 indicate item-level missing responses.

## Data Availability

The datasets analyzed in this study can be found in Mendeley Data (https://data.mendeley.com/datasets/rb47phgbyp/1; accessed on 4 March 2026).

## Supplementary Materials

The following supporting information can be downloaded at: https://www.mdpi.com/article/10.3390/healthcare14091170/s1, File S1: Sociodemographic and clinical questionnaire; File S2: Educational services form; File S3: Mental health services form.

## Abbreviations

The following abbreviations are used in this manuscript:

SUS	Unified Health System
CAPS	Psychosocial Care Center
CAPSi	Child and Adolescent Psychosocial Care Center
RAPS	Psychosocial Care Network
CER	Specialized Rehabilitation Center
UBS	Primary Health Care Unit
AEE	Specialized Educational Support Service
PNPS	National Health Promotion Policy

## References

[1] American Psychiatric Association (2022). Diagnostic and Statistical Manual of Mental Disorders.

[2] Brignell A., Harwood R.C., May T., Woolfenden S., Montgomery A., Iorio A., Williams K. (2022). Overall prognosis of preschool autism spectrum disorder diagnoses. Cochrane Database Syst. Rev..

[3] Lord C., Charman T., Havdahl A., Carbone P., Anagnostou E., Boyd B., Carr T., de Vries P.J., Dissanayake C., Divan G. (2022). The Lancet Commission on the future of care and clinical research in autism. Lancet.

[4] Hume K., Steinbrenner J.R., Odom S.L., Morin K.L., Nowell S.W., Tomaszewski B., Szendrey S., McIntyre N.S., Yücesoy-Özkan S., Savage M.N. (2021). Evidence-based practices for children, youth, and young adults with autism: Third generation review. J. Autism Dev. Disord..

[5] Maddox B.B., Dickson K.S., Stadnick N.A., Mandell D.S., Brookman-Frazee L. (2021). Mental health services for autistic individuals across the lifespan: Recent advances and current gaps. Curr. Psychiatry Rep..

[6] Shah R.P., Kunnavakkam R., Msall M.E. (2013). Pediatricians’ knowledge, attitudes, and practice patterns regarding special education and individualized education programs. Acad. Pediatr..

[7] Farmer J.E., Clark M.J., Mayfield W.A., Cheak-Zamora N., Marvin A.R., Law J.K., Law P.A. (2014). The relationship between the medical home and unmet needs for children with autism spectrum disorders. Matern. Child. Health J..

[8] Patel V., Kieling C., Maulik P.K., Divan G. (2013). Improving access to care for children with mental disorders: A global perspective. Arch. Dis. Child..

[9] Divan G., Bhavnani S., Leadbitter K., Ellis C., Dasgupta J., Abubakar A., Elsabbagh M., Hamdani S.U., Servili C., Patel V. (2021). Annual Research Review: Achieving universal health coverage for young children with autism spectrum disorder in low- and middle-income countries: A review of reviews. J. Child. Psychol. Psychiatry.

[10] Anthony C.S., Agbo C.E., Ogieuhi I.J., Ajekiigbe V.O., Agu M.C., Ayomide J.H., Oyetola E.O., Adejumo T.P., Umenzeakor K.H., Akinmeji O. (2026). Caregiving for children with autism in Africa: A scoping review of socioeconomic impact with a call for intersectoral collaboration. Eur. Child. Adolesc. Psychiatry.

[11] Hurt L., Langley K., North K., Southern A., Copeland L., Gillard J., Williams S. (2019). Understanding and improving the care pathway for children with autism. Int. J. Health Care Qual. Assur..

[12] Donoso-Estay L., Villagra Bravo C., López V. (2025). Systematizing community-based health services for autistic individuals: A municipal model from Chile. Int. J. Equity Health.

[13] World Health Organization (2008). Closing the Gap in a Generation: Health Equity Through Action on the Social Determinants of Health.

[14] Paula C.S., Cukier S., Cunha G.R., Irarrázaval M., Montiel-Nava C., Garcia R., Rosoli A., Valdez D., Bordini D., Shih A. (2020). Challenges, priorities, barriers to care, and stigma in families of people with autism: Similarities and differences among six Latin American countries. Autism.

[15] Portolese J., Bordini D., Lowenthal R., Zachi E.C., de Paula C.S. (2017). Mapeamento dos serviços que prestam atendimento a pessoas com transtornos do espectro autista no Brasil. Cad. Pos-Grad. Disturb. Desenvolv..

[16] Araripe B., Montiel-Nava C., Bordini D., Cunha G.R., Garrido G., Cukier S., Garcia R., Rosoli A., Valdez D., Caetano S.C. (2022). Profile of service use and barriers to access to care among Brazilian children and adolescents with autism spectrum disorders. Brain Sci..

[17] Ceballos G.Y., Paula C.S., Ribeiro E.L., Santos D.N. (2019). Child and adolescent Psychosocial Care Center service use profile in Brazil: 2008 to 2012. Braz. J. Psychiatry.

[18] Brasil Lei nº 12.764, de 27 de Dezembro de 2012. Institui a Política Nacional de Proteção dos Direitos da Pessoa com Transtorno do Espectro Autista. https://www.planalto.gov.br/ccivil_03/_ato2011-2014/2012/lei/l12764.htm.

[19] Brasil M.d.S. (2014). Diretrizes de Atenção à Reabilitação da Pessoa Com Transtorno do Espectro do Autismo (TEA).

[20] Ministério da Saúde (Brasil). Secretaria de Atenção à Saúde. Departamento de Atenção Especializada e Temática (2015). Linha de Cuidado Para a Atenção às Pessoas Com Transtornos do Espectro do Autismo e Suas Famílias na Rede de Atenção Psicossocial do Sistema Único de Saúde.

[21] Nunes D.R.P., Schmidt C. (2019). Educação especial e autismo: Das práticas baseadas em evidências à escola. Cad. Pesqui..

[22] Yusoff M.S.B. (2019). ABC of content validation and content validity index calculation. Educ. Med. J..

[23] Fleiss J.L., Levin B., Paik M.C. (2003). Statistical Methods for Rates and Proportions.

[24] Fulceri F., Gila L., Caruso A., Micai M., Romano G., Scattoni M.L. (2023). Building bricks of integrated care pathway for autism spectrum disorder: A systematic review. Int. J. Mol. Sci..

[25] Lima R.C., Couto M.C.V., Delgado P.G.G., Oliveira B.D.C. (2014). Indicadores sobre o cuidado a crianças e adolescentes com autismo na rede de CAPSi da região metropolitana do Rio de Janeiro. Physis.

[26] Adugna M.B., Nabbouh F., Shehata S., Ghahari S. (2020). Barriers and facilitators to healthcare access for children with disabilities in low- and middle-income sub-Saharan African countries: A scoping review. BMC Health Serv. Res..

